# Is uncertain vulvovaginal candidiasis a marker of vulvodynia? A study in a Dutch general practice research database

**DOI:** 10.3399/bjgpopen17X100905

**Published:** 2017-05-31

**Authors:** Peter Leusink, Daphne van Moorsel, Hans Bor, Gé A Donker, Peter Lucassen, Doreth Teunissen, Ellen Laan, Antoine Lagro-Janssen

**Affiliations:** 1 GP, Department of Primary and Community Care, Unit Gender & Women’s Health, Radboud University Medical Centre, Nijmegen, The Netherlands; 2 Senior Medical Student, Department of Primary and Community Care, Unit Gender & Women’s Health, Radboud University Medical Center, Nijmegen, The Netherlands; 3 Researcher, Department of Primary and Community Care, Unit Gender & Women’s Health, Radboud University Medical Center, Nijmegen, The Netherlands; 4 GP & Researcher, Primary Care Database, NIVEL, Utrecht, The Netherlands; 5 GP & Researcher, Department of Primary and Community Care, Unit Gender & Women’s Health, Radboud University Medical Center, Nijmegen, The Netherlands; 6 GP & Researcher, Department of Primary and Community Care, Unit Gender & Women’s Health, Radboud University Medical Center, Nijmegen, The Netherlands; 7 Psychologist & Professor of Sexology, Department of Sexology and Psychosomatic Obstetrics and Gynaecology, Academic Medical Center, University of Amsterdam, Amsterdam, The Netherlands; 8 Professor of Gender & Women's Health, Department of Primary and Community Care, Unit Gender & Women’s Health, Radboud University Medical Center, Nijmegen, The Netherlands

**Keywords:** general practice, vulvodynia, vulvovaginal candidiasis, clinical decision-making, differential diagnosis, uncertainity

## Abstract

**Background:**

A recent Dutch study in general practice showed a clear relationship between the diagnosis of vulvovaginal candidiasis (VVC) and symptoms suggestive of provoked vulvodynia (PVD). PVD accounts for the largest group of vulvar pains, but is often not recognised by GPs.

**Aim:**

To investigate whether diagnostic uncertainty about VVC in general practice could also point to the diagnosis of PVD, and whether and how this diagnostic uncertainty affects management.

**Design & setting:**

An observational study in 2014 in Dutch general practices of the NIVEL Primary Care Database.

**Method:**

Women with an uncertain diagnosis of VVC were distinguished from those with certain VVC based on the occurrence of recurrent episodes and persisting complaints, despite treatment. Factors known to be associated with PVD were hypothesised to be more prevalent in women with uncertain VVC. Data on symptom management by GPs were collected.

**Results:**

In total 7066 women with VVC or uncertain VVC were included. Uncertain VVC was found to account for 28% of these patients. Compared to VVC, the group uncertain VVC included significantly more women with female genital symptoms, tiredness, irritable bowel syndrome (all *P*<0.001), feeling anxious, reduced sexual desire, depressive disorder, relationship problems, and micturition symptoms (all *P*<0.05). Compared to VVC, the group uncertain VVC included significantly higher mean numbers of telephone consultations (*P*<0.001), more referrals to gynaecology (*P* = 0.009), and higher mean numbers of prescriptions per patient (*P*<0.001).

**Conclusion:**

This study's findings indicate that uncertain VVC could be a marker of PVD. GPs might reconsider their diagnostics and management when women present recurrent and persistent vulvovaginal complaints, especially if accompanied by dyspareunia, functional syndromes, micturition symptoms, and psychological conditions.

## How this fits in

Frequently, GPs are dealing with vulvovaginal complaints for which a diagnosis is not clear. Generally, mostly VVC is being considered. After excluding infections and dermatoses GPs might consider the diagnosis of PVD when women present recurrent and persistent vulvovaginal complaints, especially if accompanied by dyspareunia, functional syndromes, micturition symptoms, and psychological conditions.

## Introduction

Vulvovaginal complaints are frequently presented in general practice. Approximately 25 per 1000 female patients per year visit their GP with VVC.^1^ Dutch GPs reported 106 consultations concerning vaginal candidiasis per practice per year.^2^ About 25–30% of women with vulvovaginal complaints remain without a microbiologically explained diagnosis, even after careful evaluation.^3–6^ One reason may be that most GPs find it difficult to diagnose vulvovaginal complaints, perhaps leading to an overestimation of VVC. A Dutch study showed that GPs do not perform the examinations required to confirm their putative diagnosis, despite the recommendations given by the Dutch College of GPs (DCGP's) Guideline on Vaginal Discharge.^1,2^ Another reason for the apparently difficulty in diagnosing of VVC might be confusion with another diagnosis, for example, provoked vulvodynia (PVD).

PVD is the most common cause of vulvar pain, which is defined as ‘vulvar pain of at least 3 months duration, without clear identifiable cause, which may have potential associated factors.^7^ PVD and VVC have partially cohering symptoms. Besides white crumbling discharge, criteria for VVC involve vaginal itching and the appearance of redness of the vulva. Vaginal burning —﻿ often being mistaken for itching —﻿ and redness of the vulva, although not obligatory, appear to be symptoms of PVD as well. Both VVC and PVD can produce symptoms of irritation and tenderness in the vestibule. Women often describe their discomfort in other phrases than ‘pain’, including itching, burning, and a swelling sensation.^8^ Previous vaginal infections such as VVC and vaginosis have been identified as important triggers and risk factors for PVD because of changes in the vaginal flora and immunological response, leading to neuropathic pain conditions of the vulva.^8–12^ Also, the strong relationship between VVC and PVD may be explained by the possibility that women with VVC continue to have intercourse despite the vulvovaginal pain they experience during and shortly after the infection, possible leading to a vicious circle that maintains PVD.^13^ A recent retrospective cohort analysis in Dutch general practices showed a clear relationship between VVC and symptoms suggestive of PVD.^14^ It was hypothesised that the strong relation between VVC and PVD could be explained as well by the possibility that VVC was misdiagnosed, actually covering a subgroup of women with PVD. The prevalence of PVD in the general population is estimated to be between 3.1% and 15%, mostly affecting women <50 years of age.^15,16^ The annual incidence in the general population is estimated to be 3.1%.^17^

Since little is known about PVD in primary care and since PVD is not included in the International Classification of Primary Care (ICPC), vulvovaginal symptoms suggestive of PVD may not be recognised by the GP and consequently be registered as VVC. A lack of training in recognising PVD among physicians could also play a role in this doctor’s delay.^18,19^ Another important factor is patient delay: in a large prospective study in 300 patients it took an average of 38 months after symptom-onset before women with PVD consulted a healthcare provider for the first time.^20^ PVD is associated with several chronic pain conditions and mental health problems.^14,21–24^ Moreover, in existing literature PVD is found to be associated with medically unexplained physical symptoms (MUPS) as is seen in fibromyalgia, chronic fatigue, sleep disorders, and irritable bowel syndrome.^20,21,25^ A timely diagnosis of PVD may be helpful in improving the impaired quality of life of patients and the consumption of extra medical care that is associated with PVD.^26^

The aim of this study was to investigate whether diagnostic uncertainty about VVC is a marker of PVD. The research questions were: do women with uncertain VVC differ from women with certain VVC with respect to characteristics associated with PVD and, given the doctor’s delay, do GPs differently manage certain or uncertain VVC, and if so, how?

## Method

A retrospective analysis of women with VVC and uncertain VVC in a representative primary care database was performed. Differences between these groups in characteristics that according to the literature were associated with PVD: female genital symptoms, psychological symptoms, MUPS, relationship problems with partner, and micturition symptoms were analysed.^14,21–24^

### Data collection

Data were collected from NIVEL, a nationally representative primary care database, which uses routinely recorded data from GPs to monitor health and utilisation of health services in a representative sample of the Dutch population. In this database GPs record all diagnoses and proceedings according to the ICPC classification, during or immediately after the consultation. Diagnostic and management data were used of women aged 10–50 years who were seen for vulvovaginal complaints by their GP between 1 January 2014 and 1 January 2015. The following information was extracted from the electronic medical record: relevant diagnoses and symptoms, additional tests, prescriptions, type of contact, and referrals.

### VVC and uncertain VVC

VVC and uncertain VVC were defined as follows.

#### VVC

According to the DCGP's Guideline on Vaginal Discharge, a VVC (ICPC code X72) is considered very likely, when the patient's history and physical exam display itching and non-smelling, white crumbling discharge in combination with a red swollen vulva or vaginal wall.^1^ According to this Guideline a physical exam can be omitted if the patient recognises the complaints from an earlier episode of VVC. In case of doubt, a positive yeast culture or potassium hydroxide preparation showing hyphae is indicated to establish a definite diagnosis.

#### Uncertain VVC, as is defined for the purposes of this study:

- *Recurrent VVC (RVVC)*: when a patient presents with three or more episodes of VVC within a year, of which at least one is confirmed by the GP in a face-to-face consultation. The frequent recurrence of a VVC might be an indication that in one way or another the GP's management was insufficient. To prevent missing patients with RVVC in 2014, patients were traced with VVC once or twice in 2014, back to 2013.
- *Persisting VVC (PVVC)*: when a patient returns for a second consultation within one episode of VVC, suggesting that the previous consultation was insufficient. Episodes, used in the NIVEL Primary Care Database, are constructed periods for a specific diagnosis. Because a symptom-free period for VVC lasts 8 weeks before a new episode of VVC can be started, a complaint was considered persistent when a patient had consulted the GP at least two times with the same complaint within a time period of 8 weeks.

### Variables associated with PVD

Symptoms associated with PVD were selected based on the literature.^14,25^ These included female genital symptoms (painful intercourse [X04], other symptoms/complaints vagina [X15], other symptoms/complaints vulva [X16], vaginitis/vulvitis not otherwise specified [NOS] [X84]), pain symptoms (muscle pain [L18], irritable bowel syndrome [D93]), general weakness/tiredness (A04), psychological conditions (feeling anxious/nervous/tense [P01], feeling depressed [P03], reduced sexual desire [P07], depressive disorder [P76]), relationship problems (Z12), and micturition symptoms (dysuria/painful urination [U01], urinary frequency/urgency [U02], urination problems other [U05], cystitis/other infection [U71]) (Tables 1 and 2).

**Table 1.** Factors associated with provoked vulvodynia for vulvovaginal candidiasis (VVC) and uncertain VVC

	VVC, % (*n* = 5088)	Uncertain VVC, % (*n *= 1978)	*P*-value
**Female genital symptoms**			
Dyspareunia	1.1	2.1	<0.001^a^
Vaginal symptom/complaint other	6.9	10.3	<0.001^a^
Vulva symptom/complaint other	2.3	3.1	0.019^b^
Vaginitis/vulvitis not otherwise specified	12.6	16.9	<0.001^a^
**Medical unexplained symptoms**			
Weakness/tiredness in general	16.3	19.4	<0.001^a^
Muscle pain	4.2	4.8	0.186
Irritable bowel syndrome	5.3	6.9	0.001^c^
**Psychological conditions**			
Feeling anxious/nervous/tense	5.3	6.3	0.047^b^
Feeling depressed	4.0	4.7	0.107
Sexual desire reduced	0.1	0.3	0.012^b^
Depressive disorder	5.1	6.7	0.002^c^
**Relationship problem partner**	3.5	4.4	0.029^b^
**Micturition symptoms**			
Dysuria/painful urination	5.1	5.8	0.148
Urinary frequency/urgency	5.8	6.9	0.046^b^
Urination problems other	2.3	3.5	0.001^c^
Cystitis/other infection	27.3	29.6	0.015^b^

^a^ *P*<0.001. ^b^ *P* <0.05. ^c^ *P*<0.01.

**Table 2.** Consultations, referrals, and prescriptions related to the disorder in patients with vulvovaginal candidiasis (VVC) and uncertain VVC

	VVC (*n *= 5088)	Uncertain VVC (*n *= 1978)	*P*-value
**Telephone consultation**			
Patients, *n* (%)	2898 (57.0)	1643 (83.1)	<0.001^a^
Consultations per patient, mean (SD)	1.2 (1.7)	3.1 (4.1)	<0.001^a^
**Face-to-face consultation**			
Patients, *n* (%)	3524 (69.3)	1801 (91.1)	<0.001^a^
Consultations per patient, mean (SD)	1.5 (2.0)	3.9 (4.6)	<0.001^a^
**Referrals**			
Patients referred to gynaecology, *n* (%)	222 (4.4)	351 (17.7)	0.009^b^
Patients referred for diagnostics, *n* (%)	156 (3.1)	241 (12.2)	<0.001^a^
Patients with one or more referrals, *n* (%)	1246 (24.5)	995 (50.3)	0.006^b^
**Prescriptions**			
Patients that received local treatment, *n* (%)	2956 (58.1)	1130 (57.1)	0.002^b^
Patients that received oral treatment, *n* (%)	579 (11.4)	442 (22.3)	0.673
Prescription per patient, mean (SD)	1.9 (1.5)	4.1 (3.2)	<0.001^a^

^a^ *P*<0.001. ^b^ *P*<0.01.

### Analysis

Data from women diagnosed with VVC that were registered between 1 January 2014 and 1 January 2015 were analysed using the Statistical Product and Service Solutions (SPSS) (version 22). It was investigated whether factors associated with PVD and whether medical activities were more prevalent in women with uncertain VVC relative to VVC. Statistical significance was computed using the χ^2^ test where *P*<0.05 was considered significant.

## Results

### Population

Of 379 190 women aged 10–50 years, representing data collected by 227 GPs, 7066 were diagnosed with VVC (1.8%). The mean age was 35.1 years (SD = 11.4).

### Uncertain VVC

Of the patients that initially received the diagnosis VVC, 28% had uncertain VVC. Table 1 lists comorbidities associated with PVD for VVC and uncertain VVC. Compared to certain VVC, patients with uncertain VVC were diagnosed significantly more often with female genital symptoms (*P*<0.001), especially dyspareunia, vaginal symptoms/complaints, and NOS vaginitis/vulvitis. Also, patients with uncertain VVC showed a significantly higher incidence of general tiredness, irritable bowel syndrome, reduced sexual desire, depressive disorder, relationship problems, and micturition symptoms (Table 1).

### Management by the GP

Mean number of consultations per patient with certain VVC was 4.3 (SD = 4.9). Patients that were classified as having uncertain VVC had almost three times as many consultations; that is, 11.3 (SD = 11.9, *P*<0.001) (data not shown). For uncertain VVC the mean number per patient of telephone consultations as well as face-to-face consultations, was significantly higher (Table 2). Patients with uncertain VVC were four times as likely to be referred to a gynaecologist (*P*<0.001), and received twice as many prescriptions per patient. Table 2 lists consultations, referrals, and prescriptions in patients with VVC and uncertain VVC.

## Discussion

### Summary

Many factors known to be associated with PVD, especially dyspareunia, functional syndromes, micturition symptoms and psychological conditions, were found to be significantly more prevalent in 2014–2015 in the uncertain VVC group, such as women presenting recurrent and persistent vulvovaginal complaints. This reinforces the hypothesis that uncertain VVC could be a marker of PVD. Also, the number of face-to-face and telephone consultation and the number of referrals, related to VVC or uncertain VVC, were found to be significantly higher in the women with uncertain VVC. Because uncertain VVC was found to account for a substantial part of 28% of all patients initially diagnosed with VVC, this study's findings may point, after excluding infections and dermatoses, to the existence of a subgroup of women with PVD within the group of women with uncertain VVC.

### Strengths and limitations

A strength of this study is that the data included a large number of patients and their episodes, and that the study was performed in a GP population representative of the general Dutch population.^27^ The GPs affiliated with NIVEL Primary Care Database are motivated to perform adequate ICPC coding.

However, as no ICPC code for PVD is available, only indirect measures of PVD were available of complaints or disorders known to be related to PVD. They lack specificity, however, as some of these nonspecific complaints may also occur in case of lichen sclerosus or eczema, therefore possibly overestimating the hypothesised relation between uncertain VVC and PVD.

Using RVVC as a subtype of uncertain VVC might overestimate this relation as well because RVVC can in fact be caused by a candida infection, especially in case of comorbidity such as diabetes mellitus, reduced immunity status, and use of immunity-decreasing drugs, for example antirheumatic and chemotherapeutic drugs, corticosteroids, and antibiotics. Nevertheless, it should be emphasised that one-third to one-half of RVVC cases have no clear cause.^28^

On the other hand, the existence of a subgroup of PVD in the group VVC may also be underestimated rather than overestimated. These data show a very low number of women with sexual complaints, contrary to robust findings in the literature showing that in women with PVD reduced sexual desire and response is common.^25^ This might indicate that GPs probably did not take a sexual history adequately.

In the past PVD was described as vulvar vestibulitis syndrome or focal vulvitis, both with the misleading suffix of '–itis'. It is possible that PVD was registered by GPs under the ICPC code vaginitis/vulvitis (NOS), which in this study indeed was found to be significantly more prevalent in the group of uncertain VVC. Since only general ICPC codes were accessible for research and subcodes were not, this cannot be stated with certainty.

### Comparison with existing literature

Women with uncertain VVC were twice more likely to have dyspareunia and three times more likely to have reduced sexual desire, both found to be important characteristics of PVD.^10,14,20,23,25^ The current findings that women with uncertain VVC showed a significantly higher incidence of micturition symptoms and of irritable bowel syndrome, are in line with findings related to chronic pelvic pain as one of the maintaining factors in PVD.^12,17,21,25^ Also, psychological conditions and relationship problems with a partner, known to be associated with PVD, are more prevalent in women with uncertain VVC.^24,29^

Uncertain VVC was found to account for 28% of all patients diagnosed with VVC, which high number does correspond to the findings of a Dutch study on the factors associated with microbiologically unexplained vaginal symptoms, which showed that 25% of all the women had symptoms of unknown aetiology.^6^ It shows the difficulty of diagnosing vulvovaginal complaints by GPs, as was demonstrated previously among Dutch GPs.^2^ This difficulty has apparently repercussions for the management of VVC by GPs as is seen in the significantly higher healthcare use in women with uncertain VVC. While these findings may be the consequence of the definition of uncertain VVC itself, it also could be an expression of the well-known association between MUPS and high healthcare use.^30^ Given the higher number of MUPS in the uncertain VVC group, and the difficulty in diagnosing PVD as a chronic pain disorder without clear identifiable cause, the higher number of consultations and referrals may reflect the association between PVD and MUPS. A recent study showed that GPs take various characteristics like multiple MUPS, frequent and long consultations and many referrals into account when recognising MUPS in their patients.^31^ Equally, in case of uncertain VVC, GPs should be aware that the increasing number of consultations and comorbidity of MUPS could point to PVD.

It is unknown whether GPs ever consider PVD as an alternative diagnosis. Given the significantly higher mean number of prescriptions received per patient in the women with uncertain VVC (twice as many), GPs seem hold on to their initial diagnosis of VVC. This is in line with research that was conducted among a group of Dutch GPs, some years after the introduction of the DCGP's Guideline on Vaginal Discharge, showing that GPs did not perform the examinations required to confirm their putative diagnosis of VVC, leading to wrong diagnoses and maltreatment with antimycotics.^2^

This study's results also demonstrate that despite requesting for diagnostics more often among women with uncertain VVC, GPs referred more often to a gynaecologist as well. This is in line with a study in patients with sexual complaints attending Dutch GPs, showing that women with a sexual dysfunction were more often referred to secondary care services than were male patients with a sexual dysfunction.^32^ If PVD were to be considered as a diagnosis, a referral to a pelvic floor physical therapist or sexologist (or sexual and relationship therapist) rather than a gynaecologist would be indicated, as is stated by the DCGP's Guideline on Sexual Complaints.^33^ Other national guidelines might be consulted to apply the referring policy correctly. Does the referral policy of the GPs in this study suggest that they do not consider any alternative diagnosis when women’s vulvovaginal complaints persist? This hypothesis would be consistent with studies that show that vulvodynia is underestimated and not recognised by GPs.^30,34^

### Implications for research and practice

It is important for GPs to evaluate their diagnostics and management when persisting genital complaints, dyspareunia, and PVD associated factors are present in a patient initially diagnosed as VVC. Rather than doing more of the same, in case of uncertainty about VVC, after excluding infections and dermatoses, the GP might consider PVD, especially in situations of RVVC and PVVC.

Since an adequate diagnostic approach is needed for recognising PVD, more insight into the facilitating and limiting factors in the diagnostic process of GPs dealing with women with vulvovaginal complaints, is recommended. Learning from the way GPs diagnose and manage MUPS could be helpful. 

Many factors known to be associated with PVD were found to be significantly more prevalent in the uncertain VVC group. Diagnostic uncertainty about VVC could point to the diagnosis PVD. GPs might reconsider their diagnostics and management when persistent and recurrent genital complaints, dyspareunia, medical unexplained symptoms, micturition symptoms, and psychological conditions are present in a patient initially diagnosed as VVC.
